# Liver test pattern and histologic bile duct injury do not predict response to treatment of immunotherapy hepatotoxicity

**DOI:** 10.1097/HC9.0000000000000922

**Published:** 2026-03-26

**Authors:** Benjamin Rabin, Michael Li, Jordan S. Sack, Isaac J. Benque, Stephen D. Zucker

**Affiliations:** 1 Department of Internal Medicine, Brigham and Women’s Hospital, Boston, Massachusetts, USA; 2 Division of Gastroenterology and Hepatology, University of California, San Francisco, San Francisco, California, USA; 3 Division of Gastroenterology, Hepatology and Endoscopy, Brigham and Women’s Hospital, Boston, Massachusetts, USA; 4 Department of Internal Medicine, University of California, San Francisco, San Francisco, California, USA

**Keywords:** immune-checkpoint inhibitor cholangitis, immune-checkpoint inhibitor hepatotoxicity, immune-mediated adverse event, immunotherapy

## Abstract

**Background and Aims:**

Immune checkpoint inhibitor (ICI) cholangitis is an uncommon immune-related adverse event affecting the biliary ducts that is considered distinct from ICI hepatitis. This study examines whether the liver injury pattern or presence of histologic bile duct damage determines response to immunosuppression.

**Methods:**

This multicenter retrospective cohort study includes 298 consecutive patients with cancer who received immunotherapy between 2010 and 2021 and were originally classified as grade 3 or higher ICI-related hepatitis [alanine aminotransferase (ALT) ≥ 200 U/L]. Response to immunosuppression was stratified by pattern of liver injury (hepatocellular, cholestatic, or mixed) as defined by *R*-value, the presence of histologic bile duct damage, and the Drug-Induced Liver Injury Network (DILIN) severity score. The primary outcome was time to normalization of ALT (≤40 U/L). Secondary outcomes included time to grade 1 liver injury (ALT ≤ 100 U/L), need for additional immunosuppression, hospitalization, and death.

**Results:**

The pattern of liver injury was hepatocellular in 60%, cholestatic in 13%, and mixed in 27%. All patients received corticosteroids as first-line therapy. There was no difference in corticosteroid dose, need for an additional immunosuppressive agent, time to normalization of ALT, or time to grade 1 liver injury between groups. Outcomes were similar among patients with or without histologic bile duct damage and between those with a DILIN severity score of 3+ versus a lower score.

**Conclusions:**

The response of high-grade ICI hepatotoxicity to standard immunosuppression is similar regardless of the pattern of liver injury or the presence of bile duct damage.

## Graphical abstract

**Figure:**
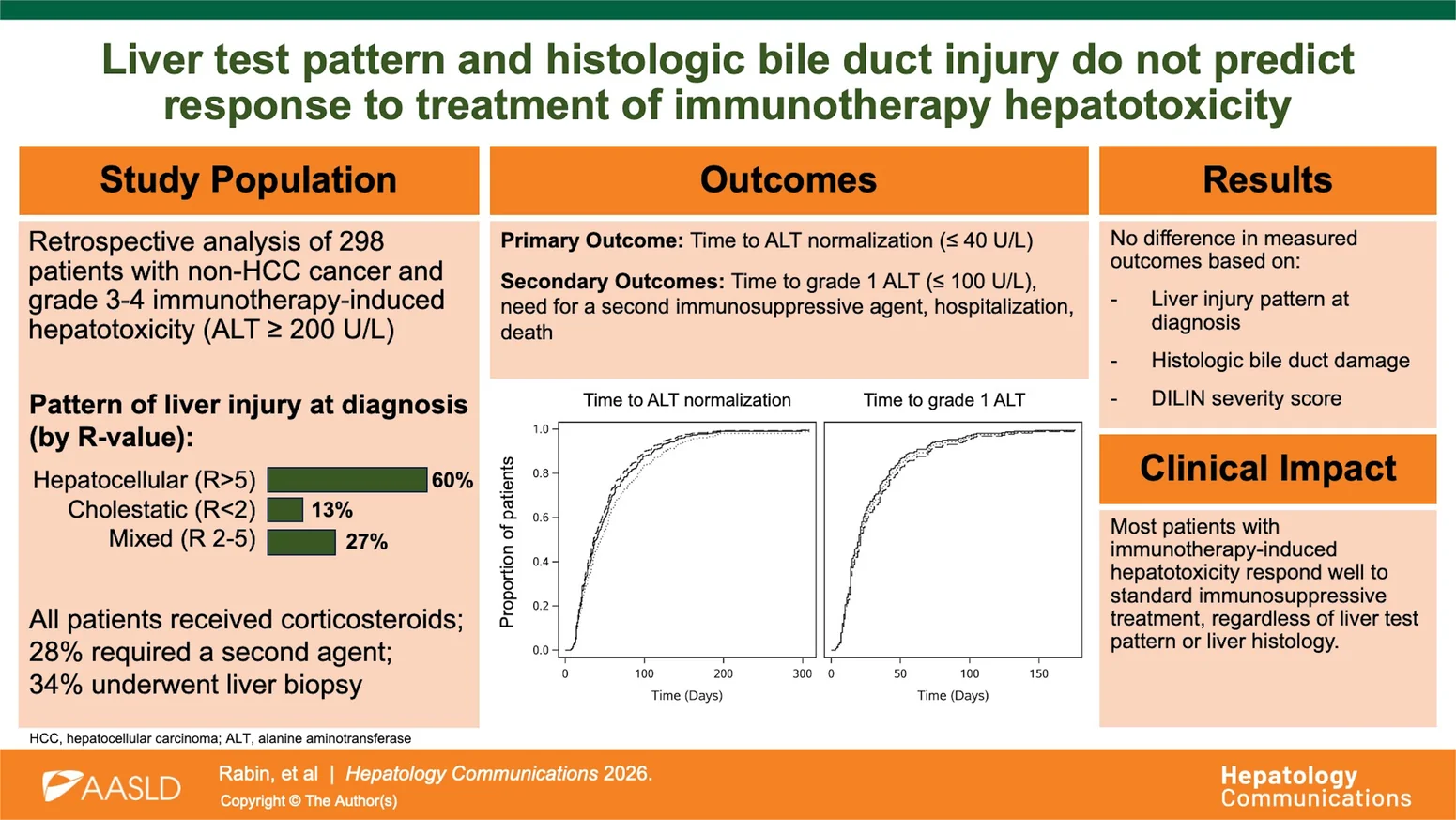

## INTRODUCTION

Immune checkpoint inhibitor (ICI) hepatotoxicity is an immune-related adverse effect (irAE) that typically manifests as asymptomatic elevations in serum alanine (ALT) and aspartate (AST) aminotransferases and responds well to ICI withdrawal and immunosuppressive therapy.^1^ Corticosteroids are considered standard first-line treatment for patients who develop grade 3 elevations in ALT from ICI therapy, with mycophenolate mofetil (MMF) and/or tacrolimus generally reserved for refractory cases.^2^^–^^5^ There is emerging consensus that ICI cholangitis represents an uncommon liver irAE that is distinct from ICI hepatitis, in which the predominant feature is injury to the small and/or large bile ducts.^6^^,^^7^ However, the precise incidence of ICI cholangitis is unclear, as studies have employed inconsistent diagnostic criteria (eg, cholestatic liver tests, histologic bile duct injury, and/or biliary abnormalities on imaging).^8^^–^^13^ While ICI hepatitis has a favorable prognosis,^14^ ICI cholangitis is considered to be more refractory to immunosuppression with increased morbidity and mortality.^6^^,^^7^^,^^12^^,^^15^^–^^18^

To better define the incidence and treatment responsiveness of ICI cholangitis, we analyzed a large retrospective cohort of patients originally classified as having grade 3 or higher ICI hepatitis to determine whether the pattern of liver enzymes or the presence of histologic bile duct injury correlates with outcome.

## METHODS

### Patient data and study design

This multicenter retrospective cohort study includes consecutive patients (excluding those with hepatocellular carcinoma) followed at the Dana–Farber/Brigham and Women’s Hospital (BWH) and Mass General Hospital (MGH) Cancer Centers between 2010 and 2020, and at the University of California San Francisco (UCSF) Medical Center between 2015 and 2021, who received immunotherapy complicated by grade 3 (≥200 U/L; 5 times the upper limit of normal) or grade 4 (≥800 U/L; 20 times the upper limit of normal) ALT elevations based on Common Terminology Criteria for Adverse Events (CTCAE) v5.0.^19^^,^^20^ Patients with alternate explanations for liver test elevations (as assessed by individual chart review), including other medications known to cause drug-induced liver injury, bile duct obstruction, or viral hepatitis were excluded, as previously described.^19^^,^^20^ The study was performed in accordance with the Declarations of Helsinki and Istanbul, and was approved by the UCSF, Dana–Farber, and Mass General Brigham institutional review boards (IRB). Informed consent was not required by the IRB, as this was a retrospective analysis, and all identifying information was carefully protected and removed from the dataset. Patients were not involved in the design, conduct, or reporting of this study. This manuscript was prepared in accordance with STROBE, http://links.lww.com/HC9/C271, reporting guidelines for cohort studies.^21^

### Patient stratification

Liver injury pattern was determined by the *R*-value calculated from liver tests obtained at the time of ICI hepatotoxicity diagnosis. As per ACG guidance,^22^ *R*-values >5 were classified as hepatocellular, <2 as cholestatic, and between 2 and 5 as a mixed pattern of liver injury. Patients were also categorized based on Drug-Induced Liver Injury Network (DILIN) severity scores of 1+ (mild), 2+ (moderate), or 3+ (moderate to severe).^23^ No patient met DILIN 4+ criteria or higher. In a sensitivity analysis, patients also were stratified according to the International DILI Expert Working Group (IEGW) scores of 1 (mild), 2 (moderate), or 3 (severe);^24^ however, no patient met criteria for an IEGW score of 3 or higher when groups were assigned based on liver tests at the time of ICI hepatotoxicity diagnosis. Patients who underwent liver biopsy were stratified by the presence of histologic bile duct damage (defined as bile duct inflammation, bile duct injury, and/or bile duct loss) as determined by experienced hepatopathologists.

### Primary and secondary outcomes

Immunotherapy was discontinued, and corticosteroids were administered to all patients as first-line therapy for grade 3 or higher ICI hepatotoxicity, in accordance with standard institutional practice. Corticosteroid dosing and route of administration, as well as the decision to administer an additional immunosuppressant, were at the discretion of the treating clinicians. Data regarding the administration of ursodeoxycholic acid (UDCA) were available for patients with cholestatic and mixed liver injury. The primary outcome was time to normalization of ALT (≤40 U/L). Secondary outcomes were time to improvement to grade 1 ALT (≤100 U/L), the need for a second immunosuppressive agent (termed steroid refractory), hospitalization, and death. In the sub-analysis, “slow responders” were defined as patients where time to ALT normalization or improvement exceeded 100 days, which represented 1 standard deviation (SD) beyond the mean time to ALT normalization for the entire cohort.

We performed additional sensitivity analyses that compared time to alkaline phosphatase (ALP) normalization (ALP ≤ 120 U/L) or improvement to grade 1 (ALP ≤ 300 U/L) among patients with cholestatic or mixed liver injury pattern versus time to ALT normalization or improvement for patients with a hepatocellular pattern of injury. We also compared secondary outcomes for ALP “slow responders” (ALP improvement exceeding 100 d), and for patients in whom the improvement in ALT or ALP exceeded 180 days, in accordance with the prior definition of “chronic DILI.”^14^

### Demographic data

Clinical data included age, sex, ethnicity, body mass index (BMI), alcohol use (yes or no), smoking (current, former, or non-smoker), and history of prior liver disease as determined by available diagnosis codes, imaging, and/or histology. Pertinent oncologic variables included cancer type, stage 4 cancer (yes or no), liver metastases (yes or no), and history and nature of prior irAE. In patients who previously received immunotherapy, the causative agent(s) were considered those received closest to the time of ICI hepatotoxicity diagnosis. Relevant laboratory variables included AST, ALT, ALP, total bilirubin, INR, creatinine, lymphocyte, and platelet count. Additional data for patients with mixed and cholestatic injury included laboratory results [eosinophil count, gamma-glutamyl transferase (GGT), conjugated bilirubin] and imaging findings [magnetic resonance cholangiopancreatography (MRCP)]. Laboratory data were obtained at 3 time points: (1) baseline (closest results before ICI initiation), (2) time of ICI hepatotoxicity diagnosis, and (3) peak values after diagnosis. Mortality was assessed through the end of follow-up (median 512 d, range 7–2687).

### Statistical analysis

Patient characteristics are reported as means and standard deviations for continuous normal data, and median and ranges for continuous non-normal data. Chi-square tests were used to compare categorical variables across the 3 liver test patterns. When frequencies were <5, a 2-sided Fisher’s exact test was employed to compare categorical variables. Means of continuous variables were compared using one-way ANOVA, and for non-normal data using Kruskal–Wallis non-parametric tests. When data were unavailable, patients were excluded from a given analysis. All patients had ALT and ALP values at the time points of interest. One patient had missing data for total bilirubin and was excluded from analyses involving DILIN and IEGW scores.

Kaplan–Meier univariate analysis with log-rank testing was applied for time-to-event data, stratified by *R*-value, DILIN severity score, and presence of histologic bile duct damage. The start date for time-to-event analysis was the date of grade 3 or higher ALT elevation. Multivariate Cox regression analysis was used to adjust for ALT values at the time of ICI hepatotoxicity diagnosis. Patients who died before ALT normalization or improvement to grade 1 injury were censored.

In an additional sensitivity analysis, we compared rates of ALP improvement for the mixed and cholestatic groups with the rate of ALT improvement for the hepatocellular group. Time to normalization (ALP ≤ 120 U/L or ALT ≤ 40 U/L) and time to grade 1 injury (ALP ≤ 300 U/L or ALT ≤ 100 U/L) were measured from the day of peak ALP or ALT, respectively. Patients whose peak ALP was ≤300 U/L were excluded from the analysis. Cox multivariate regression analysis was performed to control for the height of ALP or ALT at the time of ICI hepatotoxicity diagnosis. Analyses were conducted using SAS Studio (SAS Institute Inc.), with significance defined as *p*<0.05. *p*-Values for multiple comparisons were adjusted using the Šidak correction when appropriate.

## RESULTS

### Baseline data

A total of 298 patients developed at least grade 3 ICI-related ALT elevations, all of whom were included.^16^ Based on the *R*-value at the time of diagnosis, 179 (60%) patients manifested a hepatocellular pattern of liver injury, 39 (13%) a cholestatic pattern, and 80 (27%) a mixed pattern (Table 1). Patients with cholestatic injury were older (*p* < 0.0001), while melanoma was more prevalent in individuals with a hepatocellular pattern (*p*=0.023), presumably because they more commonly received combination (ipilimumab/nivolumab) therapy (*p*=0.003). The remaining demographic data were similar across groups. Patients with cholestatic and mixed patterns of liver injury had modestly higher ALP levels at baseline (*p* < 0.0001), although median ALP values were within the normal range for all cohorts. All other baseline laboratory data were similar across groups.

**TABLE 1:** Baseline demographic, clinical, and laboratory variables stratified by liver injury pattern as determined by *R*-value

	Cholestatic (n=39)	Hepatocellular (n=179)	Mixed (n=80)	*p*
Age at time of ICI hepatitis diagnosis, median (range)	67 (24–90)	57 (19–88)	63 (28–91)	**<0.0001**
Female, n (%)	18 (46%)	87 (49%)	50 (63%)	0.087
Ethnicity, n (%)				
White	34 (87%)	154 (86%)	68 (85%)	0.947
Black	0 (0%)	2 (1.1%)	1 (1.3%)	
Hispanic	1 (2.6%)	9 (5%)	6 (7.5%)	
Other/unknown	4 (10%)	14 (7.8%)	5 (6.3%)	
BMI, median (range)	26 (17–37)	26 (15–46)	25 (17–47)	0.613
Smoking, n (%)				0.144
Non-smoker	24 (62%)	137 (77%)	57 (71%)	
Former smoker	2 (5.1%)	11 (6.2%)	7 (8.8%)	
Current smoker	13 (33%)	31 (17%)	16 (20%)	
Prior liver disease, n (%)				0.863
None	31 (79%)	137 (77%)	60 (75%)	
Steatosis	7 (18%)	41 (23%)	18 (23%)	
Cirrhosis	1 (2.6%)	0 (0%)	1 (1.3%)	
Alcohol use, n (%)	9 (23%)	28 (15%)	8 (10%)	0.165
Cancer type, n (%)				
Melanoma	13 (33%)	102 (57%)	39 (49%)	**0.023**
Non–small cell lung cancer	9 (23%)	9 (5.0%)	12 (15%)	
Renal cell carcinoma	7 (18%)	11 (6.2%)	7 (8.8%)	
Breast cancer	2 (5.1%)	8 (4.5%)	5 (6.3%)	
Urothelial cancer	2 (5.1%)	7 (3.9%)	3 (3.8%)	
Other^a^	6 (15%)	42 (23%)	14 (18%)	
Stage 4 cancer, n (%)	30 (77%)	146 (82%)	69 (86%)	0.430
Liver metastases, n (%)	7 (18%)	45 (25%)	22 (28%)	0.521
Other irAE, n (%)	14 (36%)	80 (45%)	34 (43%)	0.601
Dermatologic	3 (7.7%)	11 (6.2%)	6 (7.5%)	
Endocrine	4 (10%)	30 (17%)	11 (14%)	
Gastrointestinal	5 (13%)	20 (11%)	15 (19%)	
Hepatic	3 (7.7%)	8 (4.5%)	1 (1.3%)	
Musculoskeletal	0	8 (4.5%)	2 (2.5%)	
Pulmonary	1 (2.6%)	7 (3.9%)	0	
Renal	1 (2.6%)	2 (1.1%)	3 (3.8%)	
Cardiac	0	4 (2.2%)	1 (1.3%)	
Other	1 (2.6%)	10 (5.6%)	7 (8.8%)	
Causative ICI, n (%)				
Combination ipilimumab + nivolumab	7 (18%)	78 (44%)	23 (29%)	**0.003**
Pembrolizumab	18 (46%)	56 (31%)	33 (41%)	0.109
Ipilimumab	3 (7.7%)	16 (8.9%)	6 (7.5%)	
Nivolumab	5 (13%)	22 (12%)	14 (18%)	
Other PD(L)-1 monotherapy^b^	6 (15%)	7 (3.9%)	4 (5.0%)	
Baseline lab values at time of ICI initiation, median (range)				
AST (U/L)	23 (9–57)	20 (5–112)	22 (11–81)	0.171
ALT (U/L)	18 (6–47)	19 (4–94)	19 (7–92)	0.940
ALP (U/L)	94 (43–270)	70 (28–477)	81 (32–770)	**<0.0001**
Total bilirubin (mg/dL)	0.4 (0.2–1.6)	0.4 (0.1–1.7)	0.5 (0.1–1.5)	0.948
Direct bilirubin (mg/dL)	0.1 (0.1–0.7)	N/A	0.1 (0–0.4)	0.970
GGT (U/L)	148 (19–254)	N/A	25 (17–191)	0.289
INR	1.0 (0.9–1.2)	1.0 (0.9–1.2)	1.0 (1.0–1.3)	0.998
Creatinine (mg/dL)	0.9 (0.45–2.1)	0.85 (0.5–2.9)	0.8 (0.5–1.7)	0.342
Platelets (×10^3^/µL)	244 (137–879)	224 (43–748)	228 (27–493)	0.388
Neutrophils (×10^3^/µL)	3.4 (0.59–40)	3.8 (0.3–19)	4.0 (0.57–34)	0.690
Lymphocytes (×10^3^/µL)	1.0 (0.14–3.7)	1.23 (0.03–3.2)	1.3 (0.1–2.7)	0.225
Eosinophils (×10^3^/µL)	0.14 (0–0.48)	N/A	0.14 (0–1.37)	0.953

Bold p values signify a p-value meeting statistical significance (p<0.05).

^a^ Other cancer types: lymphoma, primary bone (Ewing’s sarcoma, chondrosarcoma), glioblastoma, head and neck squamous cell, small cell lung, adrenal, cystic adenoid, ependymoma, gastrointestinal and hepatobiliary (esophageal, gastric, colorectal, and pancreatic adenocarcinoma, cholangiocarcinoma), leiomyosarcoma, mesothelioma, mucoepidermoid carcinoma, ovarian adenocarcinoma, thyroid (anaplastic, medullary, papillary types), prostate, and non-melanoma cutaneous (squamous, Merkel cell).

^b^ Other PD(L)-1 therapies: durvalumab, atezolizumab, avelumab, cemiplimab, and dostarlimab.

Abbreviations: ALP, alkaline phosphatase; ALT, alanine aminotransferase; AST, aspartate aminotransferase; BMI, body mass index; GGT, gamma-glutamyl transferase; ICI, immune checkpoint inhibitor; irAE, immune-related adverse event.

By definition, ALT and ALP levels at the time of diagnosis of ICI hepatotoxicity were significantly different between the hepatocellular, cholestatic, and mixed pattern cohorts (Table 2). The median total bilirubin was slightly higher, and the lymphocyte count was mildly lower in the cholestatic cohort. The incidence of grade 4 liver injury (ALT ≥ 800 U/L and/or ALP ≥ 2400 U/L) was highest in the hepatocellular cohort (*p* < 0.0001), with no patients meeting CTCAE grade 4 criteria based on ALP values. While 10% of patients with cholestatic injury manifested total bilirubin levels >3 times the upper limit of normal (bilirubin > 3.6 mg/dL) compared with 3.8% and 2.3% of the mixed and hepatocellular groups, respectively, this difference did not reach statistical significance (*p*=0.073). Six patients (15%) with a cholestatic liver injury pattern manifested a DILIN severity score of 3+ at diagnosis compared with 3 (4%) and 9 (5%) patients in the mixed and hepatocellular groups, respectively (*p*=0.048). The IEGW criteria produced nearly identical results, with a greater proportion of patients in the cholestatic group manifesting a moderate injury (*p*=0.01). All other lab tests were similar across groups.

**TABLE 2:** Laboratory tests at the time of immunotherapy-induced hepatotoxicity diagnosis, stratified by liver injury pattern

	Cholestatic (n=39)	Hepatocellular (n=179)	Mixed (n=80)	*p*
Lab values, median (range)				
AST (U/L)	272 (94–624)	261 (53–3085)	234 (73–1498)	0.387
ALT (U/L)	259 (168–569)	376 (194–2641)	291 (200–1182)	**<0.0001**
ALP (U/L)	694 (288–2129)	117 (45–571)	296 (150–1384)	**<0.0001**
Total bilirubin (mg/dL)	1.1 (0.3–6.3)	0.6 (0.1–12.8)	0.7 (0.2–21.3)	**0.0004**
Direct bilirubin (mg/dL)	0.45 (0.1–4.3)	N/A	0.3 (0.1–17.7)	0.673
GGT (U/L)	540 (35–2096)	N/A	457 (132–4406)	0.643
INR	1.2 (0.9–1.4)	1.1 (0.9–1.4)	1.0 (0.9–1.2)	0.088
Creatinine (mg/dL)	0.95 (0.46–2.5)	0.83 (0.44–3.3)	0.81 (0.42–1.8)	0.127
Platelets (×10^3^/µL)	224 (58–856)	212 (16–529)	224 (8–443)	0.408
Neutrophils (×10^3^/µL)	4.0 (0.64–15)	4.0 (0.1–15)	3.9 (0.38–13)	0.753
Lymphocytes (×10^3^/µL)	0.62 (0.05–2.8)	1.2 (0.01–3.4)	0.95 (0.06–3.4)	**0.0009**
Eosinophils (×10^3^/µL)	0.13 (0–1.15)	N/A	0.07 (0–4.91)	0.181
Grade 4 injury (ALT ≥ 800 U/L), n (%)	0 (0%)	35 (20%)	3 (3.8%)	**<0.0001**
Total bilirubin >3.6 mg/dL, n (%)	4 (10%)	4 (2.3%)	3 (3.8%)	0.073
DILIN severity score 3+, n (%)	6 (15%)	9 (5%)	3 (4%)	**0.048**
IEGW score moderate, n (%)	7 (18%)	10 (5.6%)	3 (3.8%)	**0.01**

Bold p values signify a p-value meeting statistical significance (p<0.05).

Abbreviations: ALP, alkaline phosphatase; ALT, alanine aminotransferase; AST, aspartate aminotransferase; DILIN, Drug-Induced Liver Injury Network; GGT, gamma-glutamyl transferase; IEGW, International DILI Expert Working Group.

Findings were similar when cohorts were assigned based on *R*-values calculated from peak lab values (Supplemental Table S1, http://links.lww.com/HC9/C270). Greater proportions of patients in the cholestatic (13%) and mixed (11%) liver injury groups manifested peak total bilirubin levels that exceeded 3 times the upper limit of normal (bilirubin >3.6 mg/dL) compared with those with hepatocellular (3.4%) injury (*p*=0.017). The proportions of patients manifesting a DILIN severity score of 3+ were similar across the groups (*p*=0.058). In all, 3 (3.8%) patients in the mixed liver injury group manifested severe injury by IEGW criteria based on peak values. Greater proportions of patients in the cholestatic (18%) and mixed (18%) injury groups manifested either moderate or severe liver injury by IEGW criteria as compared with the hepatocellular (7.8%) injury group (*p*=0.036).

### Medical treatment

The time from ICI hepatotoxicity diagnosis to corticosteroid initiation, as well as the median and maximum corticosteroid doses (expressed as methylprednisolone equivalents), were similar (*p*=0.532) across groups (Table 3). Initial therapy was with IV methylprednisolone in approximately half of patients in each group (*p*=0.927), with the remaining receiving oral prednisone. The percentage of patients who were administered a second immunosuppressive agent (28% of the total cohort) was no different across groups, with mycophenolate mofetil (MMF) used in the majority (84%). The maximum dose of MMF (*p*=0.389) and time from initiation of corticosteroids to commencing MMF (*p*=0.446) were no different between groups. Adjunctive therapy with ursodeoxycholic acid (UDCA) was rarely used, with only 1 patient (2.6%) in the cholestatic group and 4 patients (5%) in the mixed liver injury group receiving the medication.

**TABLE 3:** Outcomes stratified by liver injury pattern

	Cholestatic (n=39)	Hepatocellular (n=179)	Mixed (n=80)	*p*
ALT improved to ≤40 U/L, n (%)	36 (92%)	170 (95%)	76 (95%)	0.704
Time (d) to ALT ≤40 U/L, median (range)	28 (9–196)	46 (7–308)	36 (7–180)	0.106
ALT improved to ≤100 U/L, n (%)	38 (97%)	178 (99%)	79 (99%)	0.221
Time (d) to ALT ≤100 U/L, median (range)	17 (3–142)	21 (4–176)	21 (3–132)	0.500
Liver biopsy, n (%)	13 (33%)	60 (34%)	29 (36%)	0.905
Cholangitis (n, % patients with biopsy)	4 (31%)	4 (6.7%)	4 (14%)	**0.046**
Bile duct injury/loss	9 (69%)	8 (13%)	11 (38%)	**<0.0001**
Cholangitis and/or bile duct injury/loss	10 (77%)	8 (13%)	11 (38%)	**<0.0001**
Hospitalized, n (%)	19 (49%)	102 (57%)	49 (61%)	0.432
Time (d) from diagnosis to steroid initiation, median (range)	0 (0–76)	0 (0–35)	0 (0–61)	0.504
Steroid dose at diagnosis (mg/kg) in MP equivalent, median (range)	1.0 (0.5–4.0)	1.0 (0.5–20.0)	1.2 (0.2–15.0)	0.532
Initial therapy with intravenous MP, n (%)	19 (49%)	92 (51%)	42 (53%)	0.927
Maximum steroid dose (mg/kg) in MP equivalent, median (range)	1.0 (0.5–5.0)	1.3 (0.5–20.0)	1.6 (0.3–15.0)	0.170
Steroid refractory, n (%)	10 (26%)	56 (31%)	18 (23%)	0.324
MMF, n (% steroid refractory)	10 (100%)	52 (93%)	14 (78%)	
Tacrolimus, n (%)	0	6 (11%)	3 (17%)	
Azathioprine, n (%)	0	1 (1.8%)	1 (5.6%)	
Abatacept, n (%)	0	1 (1.8%)	1 (5.6%)	
IVIG, n (%)	0	2 (3.6%)	0	
Time (d) from steroid initiation to addition of MMF, median (range)	7 (2–66)	9 (0–164)	12 (3–64)	0.446
MMF maximum dose (mg daily), median (range)	2000 (1000–2000)	2000 (1000–3000)	2000 (500–3000)	0.389
Ursodeoxycholic acid (UDCA), n (%)	1 (2.6%)	N/A	4 (5%)	1.000
UDCA maximum dose (mg/day), median (range)	900 (900–900)	N/A	600 (300–900)	0.263
Death during follow-up (all-cause), n (%)	15 (38%)	69 (39%)	43 (54%)	0.063
Time (d) to death, median (range)	220 (10–1617)	372 (16–1470)	249 (7 – 1774)	0.206
Death before ALT ≤40 U/L, n (%)	2 (5.1%)	6 (3.4%)	3 (3.8%)	0.747
Death before ALT ≤100 U/L, n (%)	0 (0%)	1 (0.6%)	0 (0%)	1.000
Duration (d) of follow-up, median (range)	322 (10–2245)	590 (16–2665)	388 (7–2687)	**0.012**

Bold p values signify a p-value meeting statistical significance (p<0.05).

Abbreviations: ALT, alanine aminotransferase; IVIG, intravenous immunoglobulin; MMF, mycophenolate mofetil; MP, methylprednisolone; UDCA, ursodeoxycholic acid.

### Outcome—Time to biochemical resolution/improvement

Time to ALT normalization (Figure 1A) and to grade 1 ALT elevation (Figure 1B) were similar, regardless of the pattern of liver injury. While ALT levels were higher in those with a hepatocellular liver injury pattern, correction for ALT at diagnosis by multivariate Cox regression did not meaningfully alter the findings (Figures 1C, D). Patients across all groups experienced similar rates of ALT normalization and hospitalization (*p*=0.432), and had an equal likelihood of requiring a second immunosuppressive agent (*p*=0.324) (Table 3). On sensitivity analysis examining only those with steroid-responsive disease, time to ALT normalization was no different across groups.

**FIGURE 1:**
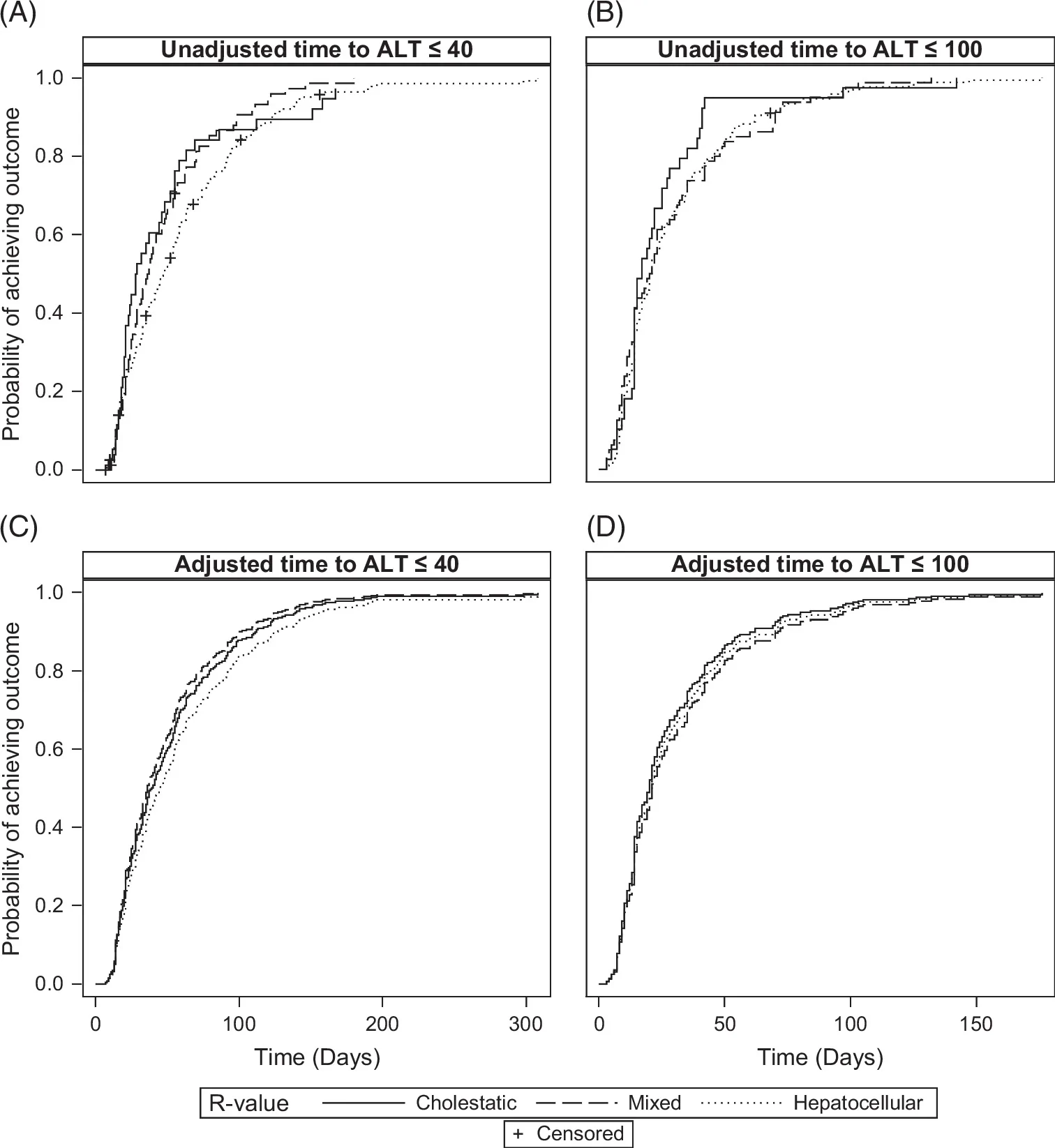
Survival analysis by liver injury pattern as determined by *R*-value for: (A) time to ALT ≤ 40 U/L (*p*=0.106); (B) time to ALT ≤ 100 U/L (*p*=0.500); (C) time to ALT ≤ 40 U/L, adjusted for ALT at the time of ICI hepatotoxicity diagnosis (*p*=0.169); (D) time to ALT ≤ 100 U/L adjusted for ALT at the time of ICI hepatotoxicity diagnosis (*p*=0.112). Solid line: cholestatic injury pattern; dashed line: mixed injury pattern; dotted line: hepatocellular injury pattern. Patients who died before improvement to ALT ≤ 40 U/L or ≤ 100 U/L were censored from analysis. Abbreviations: ALT, alanine aminotransferase; ICI, immune checkpoint inhibitor.

We performed additional sensitivity analyses comparing time from peak ALT to ALT normalization or improvement to grade 1 for all groups versus time from peak ALP to ALP normalization or improvement to grade 1 for the mixed and cholestatic injury groups (Supplemental Table S2, http://links.lww.com/HC9/C270). ALP normalization (≤ 120 U/L) was slower in the group with cholestatic liver injury as compared with ALT normalization in the hepatocellular group and ALP normalization in the mixed injury group, even after adjusting for the height of ALT and ALP at the time of ICI hepatotoxicity diagnosis (*p* < 0.0001) (Supplemental Figure S1A, http://links.lww.com/HC9/C270). On the other hand, patients with cholestatic liver injury exhibited significantly more rapid improvement to grade 1 (ALP ≤ 300) compared with patients with hepatocellular (grade 1 ALT) injury pattern (*p*=0.0003). (Supplemental Figure S1B, http://links.lww.com/HC9/C270). Rates of ALP improvement to grade 1 (≤300) among patients with cholestatic versus those with mixed injury did not achieve statistical significance (*p*=0.192).

### Outcomes—Slow responders

In an attempt to identify whether there exists a subgroup of patients who may respond less well to treatment, we examined outcomes in patients deemed “slow responders,” defined as time from diagnosis to normalization or improvement in ALT to grade 1 of >100 days (>1 SD beyond the mean) (Supplemental Table S3, http://links.lww.com/HC9/C270). While slow responders were more likely to receive an additional immunosuppressive agent (*p*=0.002), an equal proportion of patients with each pattern of liver injury were slow to resolve ICI hepatotoxicity, arguing against the existence of a subset of patients within the cholestatic or mixed cohorts that were more refractory to immunosuppression.

In further sensitivity analyses, we compared outcomes in patients where time from peak ALT to normalization (≤40 U/L) exceeded 100 days with those patients where time from peak ALP to normalization (≤120 U/mL) exceeded 100 days (Supplemental Table S4, http://links.lww.com/HC9/C270). While patients in the cholestatic injury group were more likely to meet criteria for “slow response” based on ALP criteria (*p*=0.0001), rates of hospitalization and death were similar across the groups. Slow responders with hepatocellular injury were more likely to receive a second immunosuppressive agent (*p*=0.002) and to undergo liver biopsy (*p*=0.03).

Patients in the cholestatic group also were more likely to meet criteria for “chronic DILI,” defined as time from hepatotoxicity diagnosis to ALT ≤ 40 U/L (hepatocellular group) or ALP ≤ 120 (cholestatic and mixed groups) exceeding 6 months (Supplemental Table S5, http://links.lww.com/HC9/C270). Patients with chronic DILI and a hepatocellular or mixed injury pattern were more likely to receive an additional immunosuppression agent (*p*=0.0006). Other outcomes were similar across the 3 groups.

### Outcomes—Death

A total of 127 patients (43%) died during follow-up (Table 3). The time to death was similar across the groups (*p*=0.206). There was a trend toward a higher mortality in the mixed liver injury cohort (*p*=0.063); however, this became less significant after controlling for cancer type (melanoma vs. non-melanoma, *p*=0.270) and cancer stage (*p*=0.206). Only 1 patient (0.3%) died before ALT improvement to ≤100 U/L, while eleven (3.6%) died before ALT normalization, with no difference in the percentage across groups (*p*=0.747). No liver-related deaths occurred.

### Outcomes by severity scores

When comparing outcomes of patients with a DILIN severity score of 3+ at diagnosis versus those with a lower score (1+ and 2+ severity were pooled as only one patient manifested a score of 2+), there was no difference in time to ALT normalization (Figure 2A), ALT improvement to ≤100 U/L (Figure 2B), the need for a second immunosuppressive agent, liver biopsy, nor in the percentage or time to death (Supplemental Table S6, http://links.lww.com/HC9/C270). As hospitalization is a component of DILIN severity scoring (which could potentially skew the findings), we also applied the IEGW criteria to our cohort, yielding findings nearly identical to DILIN (Supplemental Table S6, http://links.lww.com/HC9/C270). The likelihood of hospitalization was greater among patients with IEGW moderate versus mild injury (*p*=0.002).

**FIGURE 2:**
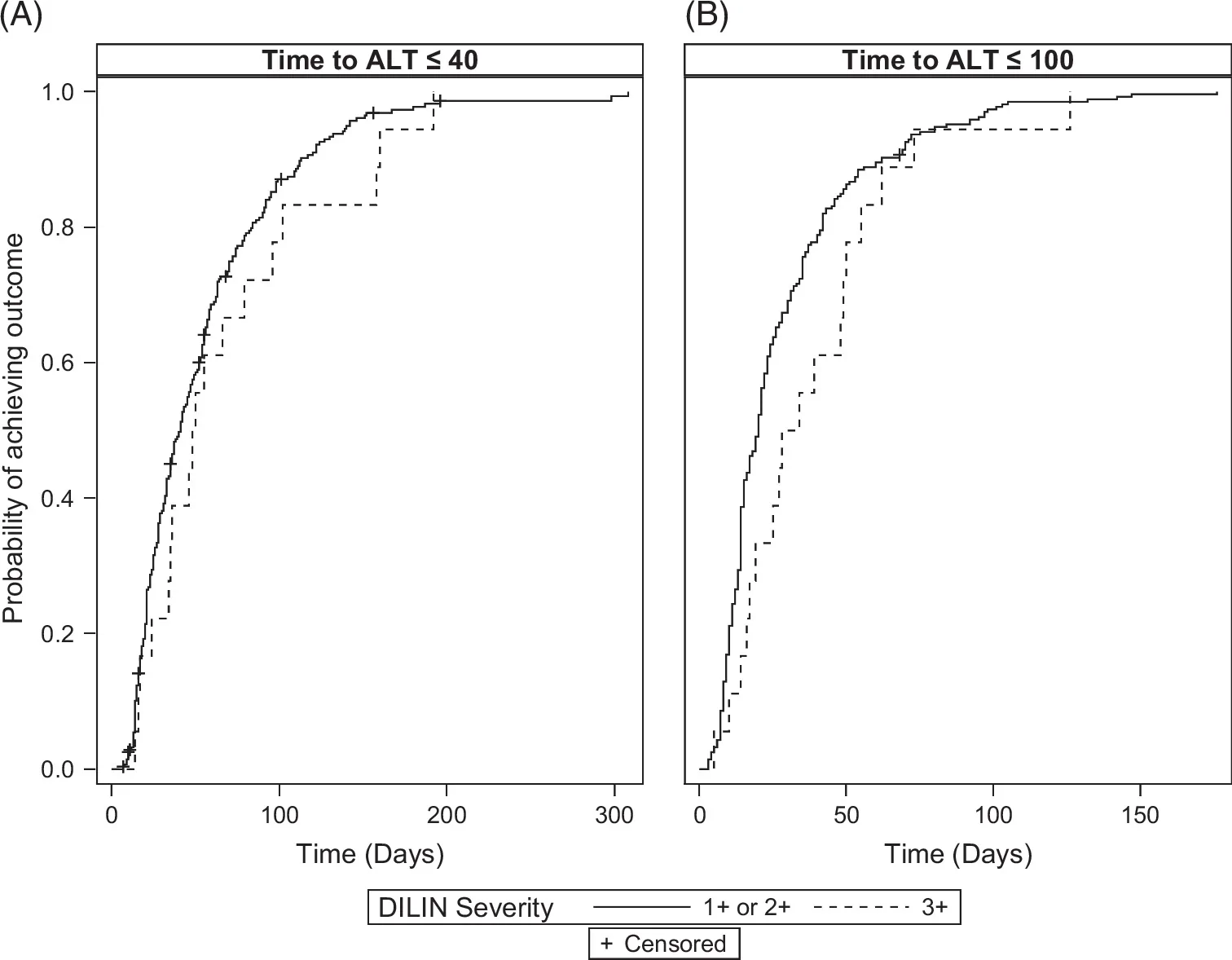
Survival analysis stratified by DILIN severity (DILIN 1+ or 2+ vs. DILIN 3+) at time of ICI hepatotoxicity diagnosis: (A) time to ALT ≤ 40 (*p*=0.254); and (B) time to ALT ≤ 100 U/L (*p*=0.148). Solid line: DILIN 1+ or 2+ based on lab tests at the time of ICI hepatotoxicity diagnosis; dashed line: DILIN 3+. Patients who died before improvement to ALT ≤ 40 U/L or ≤ 100 U/L were censored from analysis. Abbreviations: ALT, alanine aminotransferase; DILIN, Drug-Induced Liver Injury Network; ICI, immune checkpoint inhibitor.

### Outcomes by liver histology

Approximately one-third of patients in each group underwent liver biopsy. To assess whether histologic evidence of bile duct involvement was predictive of response to treatment, we compared outcomes in patients who manifested bile duct damage (defined as bile duct inflammation, bile duct injury, and/or bile duct loss) to those who did not. While the incidence of bile duct damage was significantly higher (*p* < 0.0001) in the cholestatic and mixed groups (Table 3), time to ALT normalization (Figure 3A) and to grade 1 injury (Figure 3B) were no different. Rates of hospitalization, the need for a second immunosuppressive agent, and mortality also were similar between the groups (Supplemental Table S7, http://links.lww.com/HC9/C270). The histologic findings in all patients with a cholestatic liver injury pattern who underwent liver biopsy are summarized in Supplemental Table S8, http://links.lww.com/HC9/C270.

**FIGURE 3:**
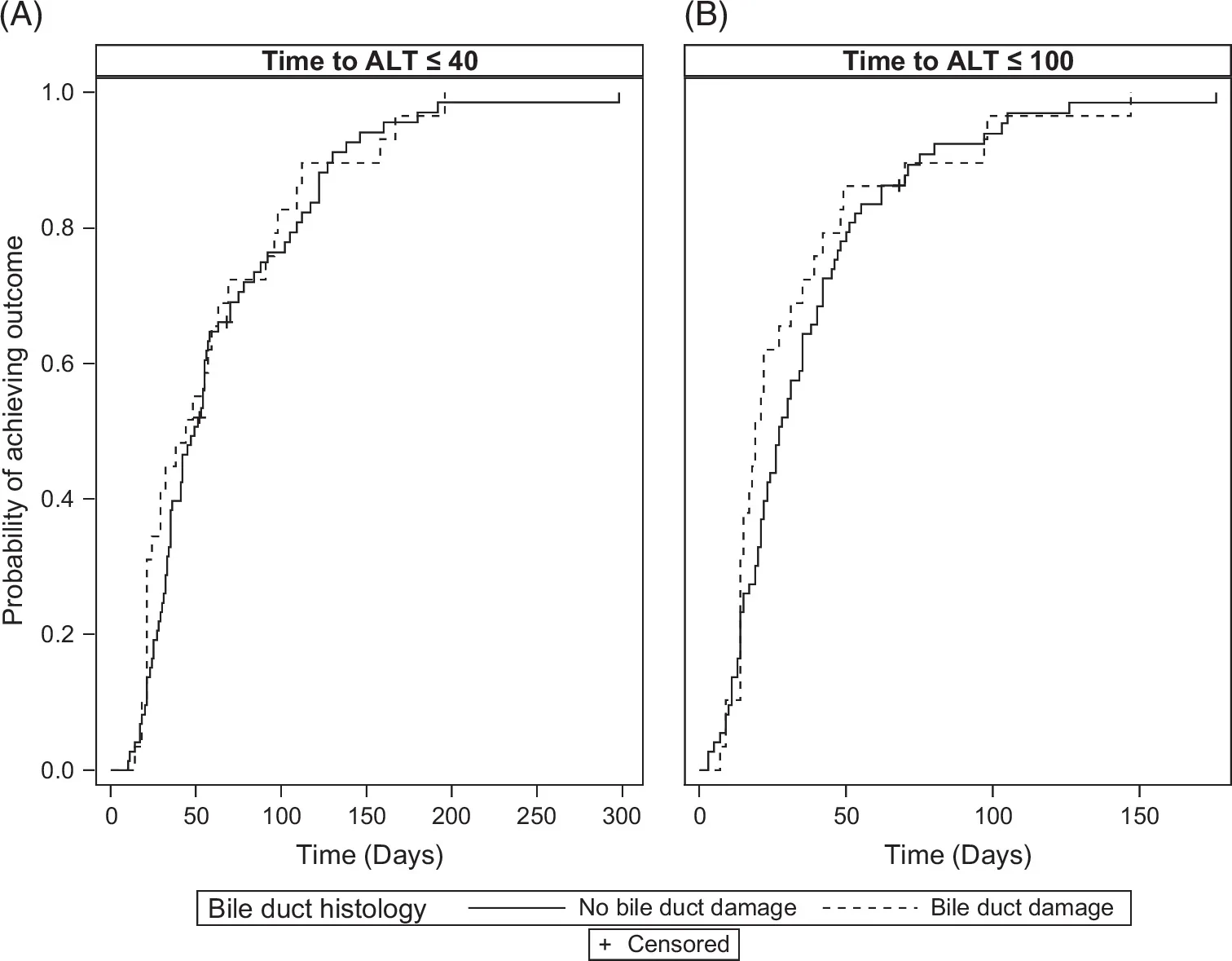
Survival analysis stratified by the presence or absence of bile duct damage on liver biopsy for: (A) time to ALT ≤ 40 (*p*=0.698) and (B) time to ALT ≤ 100 U/L (*p*=0.446). Solid line: no bile duct damage; dashed line: bile duct damage seen on liver biopsy. Patients who died before improvement to ALT ≤ 40 U/L or ≤100 U/L were censored from analysis. Abbreviation: ALT, alanine aminotransferase.

### MRCP findings

An MRCP was obtained in a minority of patients in the cholestatic (36%) and mixed (21%) liver injury groups (Supplemental Table S9, http://links.lww.com/HC9/C270). In the majority (77%), no bile duct abnormalities were noted. The proportion of patients with intrahepatic and/or extrahepatic biliary involvement on MRCP was similar between the cholestatic and mixed injury groups. The most common finding was mild biliary ductal dilatation (either intrahepatic and/or extrahepatic) that predated or occurred subsequent to the diagnosis of ICI hepatotoxicity. All of these patients underwent liver biopsy, with liver histology showing no evidence of bile duct abnormalities, and all exhibited biochemical improvement with immunosuppression. No patient manifested biliary strictures on MRCP. A single patient in the mixed injury cohort had evidence of biliary ductal thickening.

## DISCUSSION

ICI cholangitis is an uncommon irAE affecting the biliary system that appears to be distinct from ICI hepatitis.^9^^,^^25^^,^^26^ Patients with ICI cholangitis are generally considered to be more refractory to corticosteroid therapy, to exhibit slower biochemical resolution, and to be at a higher risk for progression to irreversible liver damage and death.^17^^,^^18^ Various diagnostic criteria for ICI cholangitis have been proposed, including a cholestatic pattern of liver enzymes, liver histology demonstrating bile duct injury, and/or bile duct abnormalities on imaging (eg, MRCP).^8^^–^^13^ However, as there is no accepted definition of ICI cholangitis, the incidence of this irAE is poorly defined and treatment practices vary widely.

To better understand the frequency of ICI cholangitis and responsiveness to immunosuppressive therapy, we stratified a large cohort of patients originally classified as having grade 3 or higher ICI hepatitis (ALT ≥ 200) by the calculated *R*-value for liver tests at the time of presentation. The majority (60%) of patients manifested a hepatocellular pattern of liver injury, while 13% exhibited a cholestatic pattern, and 27% a mixed pattern. This distribution mirrors prior reports by DILIN^14^ and 2 retrospective reviews of liver histology in patients with immunotherapy-induced hepatotoxicity.^27^^,^^28^ Other retrospective studies have reported higher proportions of patients with cholestatic or mixed biochemical injury patterns,^8^^,^^23^ likely due to differences in inclusion criteria. Consistent with prior studies,^8^^,^^27^ of the patients in our cohort presenting with cholestatic biochemistries, the majority (77%) exhibited histologic evidence of bile duct damage, compared with much smaller percentages of those presenting with mixed (38%) or hepatocellular injury patterns (13%). Abdominal MR imaging was obtained in a minority of patients (36% with cholestatic injury, 21% with mixed injury) with most (77%) exhibiting a normal biliary system. The most common finding was mild biliary ductal dilatation, with a single patient exhibiting bile duct thickening and none manifesting biliary strictures. These data mirror those of a prior cohort study that described bile duct findings in 18% of patients with cholestatic injury^8^ and a report that identified no cases of biliary stricture on MRI,^29^ but are discordant with a separate study describing higher rates of macroscopic biliary duct stricturing.^30^

We did not observe any difference in measured outcomes based on the pattern of liver injury at diagnosis. This included time to ALT normalization (≤40 U/L) or improvement to grade 1 (≤100 U/L), need for additional immunosuppression, hospitalization rate, or death. These findings are similar to prior reports,^1^^,^^8^^,^^14^ with the caveat that a higher proportion of patients in one study^14^ had persistent elevations in liver tests beyond 6 months. However, we speculate that study cohort had an increased preponderance of patients with underlying alcohol-associated and/or metabolic dysfunction associated steatohepatitis, as a high proportion manifested steatosis on liver histology (which is not a characteristic feature of ICI hepatotoxicity), and those who experienced protracted liver test elevations had significantly higher baseline ALT levels. Consistent with other reports,^27^ we found no difference in time to ALT normalization or improvement when comparing patients with and without histologic bile duct injury. This observation suggests that performing routine liver biopsy in patients with a high index of suspicion for ICI-induced hepatotoxicity^7^ is unwarranted.

Our a priori biochemical marker for hepatotoxicity resolution was normalization of ALT, as elevations in ALT (and/or bilirubin) form the basis for ICI discontinuation, as well as the initiation and eventual tapering of immunosuppressive medications.^3^ However, acknowledging that patients with cholestatic or mixed liver injury may exhibit more protracted ALP elevations, we also assessed the ALP response to treatment in patients with cholestatic and mixed liver injury patterns. While our findings suggest that ALP normalization is modestly more protracted in patients with a cholestatic liver injury pattern, the clinical significance of this finding is uncertain as ALP improvement to grade 1 (≤300 U/L) was actually more rapid in this same cohort. We speculate that persistent, low-grade elevations in ALP may be more reflective of progression of the underlying malignancy (eg, bony metastases, liver metastases, bile duct compression) as a consequence of discontinuing immunotherapy, or possibly effects of alternative chemotherapies, as opposed to lingering ICI hepatotoxicity. Overall, our findings support that patients with a cholestatic liver injury pattern respond well to standard immunosuppression.

The diagnostic criteria for ICI cholangitis have not been well-established.^13^ Our data suggest that, in patients with at least grade 3 ICI-related ALT elevation, neither the biochemical pattern on presentation nor the presence of bile duct injury on liver biopsy predict response to immunosuppressive therapy. If the diagnosis of ICI cholangitis is limited to individuals with bile duct injury and a poor response to immunosuppression, it appears to be a rare liver irAE, as all of the 298 consecutive patients in our cohort exhibited excellent biochemical improvement with standard therapy, and biliary abnormalities on MR imaging (when obtained) were uncommon. Although it has been proposed that the DILIN severity score is more accurate than CTCAE criteria in predicting the outcome of ICI hepatotoxicity,^19^ we observed that response to immunosuppression and outcomes were similar in patients with a DILIN severity score of 3+ versus those with lower scores (no patient in our cohort manifested a DILIN score of 4+ or higher). Findings were similar when IEGW DILI criteria were utilized.

There is minimal guidance on the most effective approach to the treatment of ICI cholangitis. Our findings support that most patients with ICI hepatotoxicity and grade 3 or higher ALT elevations improve with standard immunosuppression. We found no evidence that patients presenting with cholestatic liver tests or histologic bile duct abnormalities respond more slowly to corticosteroid treatment or are more likely to be steroid refractory. While ursodeoxycholic acid has been proposed as a potential first-line therapy, data are limited to small, observational studies.^8^^,^^29^ Since all of the patients in our study received upfront corticosteroids, and UDCA was rarely prescribed, we are unable to comment on the potential efficacy of UDCA as mono or adjunct therapy, as has been proposed by others.^29^ In the rare instance where a patient with ICI hepatotoxicity demonstrates an insufficient response to a standard immunosuppressive regimen, tocilizumab has been used with success, including those with ICI cholangitis.^12^^,^^31^

A strength of our study is the large, well-characterized multicenter cohort of patients with ICI-related hepatotoxicity. Because inclusion was based on grade 3 or higher ALT elevation, this study would inherently exclude those patients who did not reach this threshold, potentially including a subset with grade 3 or higher ALP elevation. On the other hand, as standard treatment protocols for grade 3 ICI hepatotoxicity were prevailing across all institutions, the inclusion criteria fostered consistency in therapeutic management: ICI withdrawal, first-line corticosteroids, and addition of a second agent when response was inadequate. Moreover, interpretation of ALP elevations and response to treatment can be confounded by liver metastases, bone metastases, and/or bile duct compression, particularly in the setting of immunotherapy withdrawal. Additional study limitations include the retrospective design, raising the possibility of unmeasured confounders. As this was an observational study, the decision to initiate a second-line immunosuppressive agent was at the discretion of the treating team. While we cannot exclude potential selection bias as to whether patients received a second agent or underwent liver biopsy, that rates were similar across all 3 patterns of liver injury argues against this being a major concern. Although there was no central pathology review, all participating institutions are high-volume liver centers with experienced hepatopathologists. A prior central review of liver histology in cases of ICI hepatotoxicity at BWH and MGH identified no substantial divergence from the original histologic description.^32^

In summary, our findings indicate that the majority of patients with grade 3 or higher ICI-related hepatotoxicity will respond to standard immunosuppression, regardless of the pattern of liver injury or the presence of histologic bile duct damage. These data suggest that the incidence of treatment-refractory ICI hepatotoxicity is extremely low (well under 1% of patients). While it has been argued that the DILIN severity score should supplant the CTCAE criteria due to superiority at predicting liver dysfunction and 90-day mortality,^23^^,^^33^ we found that neither the DILIN score (when 3+ or lower) nor the IEGW DILI criteria (whether mild or moderate) correlated with biochemical response to immunosuppression or other clinical outcomes. As improvement in ALT is an important determinant of whether cancer patients can resume chemotherapy or enter into therapeutic trials, the CTCAE criteria remain a reasonable guide to managing ICI hepatotoxicity.

## Supplementary Material
