# Supplementary material for: Liver test pattern and histologic bile duct injury do not predict response to treatment of immunotherapy hepatotoxicity
Source: Hepatol Commun. 2026 Mar 26;10(4):e0922. doi: 10.1097/HC9.0000000000000922 (PMC13671605; doi:10.1097/HC9.0000000000000922)
Supplement: Supplementary file 1 [file hc9-10-e0922-s001.pdf]

Supplemental Materials, Table of Contents	Page #
Supplemental Table 1: Peak laboratory tests stratified by liver injury pattern	2-3
Supplemental Table 2: Outcomes stratified by R-value based on peak alanine aminotransferase (ALT) and peak alkaline phosphatase (ALP)	4
Supplemental Table 3: Outcomes of slow responders, defined by time to ALT normalization or improvement, stratified by liver injury pattern	5
Supplemental Table 4: Outcomes of slow responders, defined by peak ALT or peak ALP values	6
Supplemental Table 5: Outcomes of ‘chronic DILI,’ defined by time to ALT or ALP normalization exceeding 6 months	7
Supplemental Table 6: Outcomes stratified by DILIN and International DILI Expert Working Group (IEGW) severity	8
Supplemental Table 7: Outcomes stratified by the presence/absence of bile duct damage on liver biopsy	9
Supplemental Table 8: Histology findings of patients with cholestatic injury who underwent liver biopsy	10-11
Supplemental Table 9: Summary of MRCP findings	12
Supplemental Figure 1: Survival analysis comparing adjusted time to ALT vs ALP normalization, stratified by liver injury pattern	13-14

Supplemental Table 1 – Peak laboratory tests (median, range) stratified by liver injury pattern through the end of follow up.

	**Cholestatic (n=39)**	**Hepatocellular (n=179)**	**Mixed (n=80)**	**P value**
**AST (U/L)**	249 (94 - 624)	297 (48 - 3085)	288 (68 - 1498)	0.479
**ALT (U/L)**	312 (168 - 775)	526 (194 - 3346)	395 (200 - 1202)	**<0.0001**
**ALP (U/L)**	791 (310 - 2129)	122 (12 - 575)	378 (155 - 1762)	**<0.0001**
**Total bilirubin (mg/dL)**	1.3 (0.3 - 6.4)	0.7 (0.1 - 12.9)	0.8 (0.2 - 21.3)	**<0.0001**
**Direct bilirubin (mg/dL)**	0.7 (0.1 - 6.3)	N/A	0.5 (0.1 - 33.5)	0.574
**GGT, (U/L)**	652 (35 - 2096)	N/A	351 (34 - 4406)	0.091
**INR**	1.2 (1.0 - 1.4)	1.1 (0.9 - 1.4)	1.0 (0.9 - 2.2)	0.316
**Creatinine (mg/dL)**	1.0 (0.4 - 2.5)	0.8 (0.5 - 3.3)	0.8 (0.4 - 2.3)	**0.037**
**Platelets ($x10^3$/uL)**	259 (60 - 856)	214 (26 - 513)	231 (10 - 443)	**0.019**
**Neutrophils ($x10^3$/uL)**	5.9 (1.9 - 21)	4.8 (0.1 - 18)	5.4 (0.6 - 22)	0.524
**Lymphocytes ($x10^3$/uL)**	0.8 (0.1 - 2.8)	1.2 (0 - 12.4)	1 (0.1 - 3.8)	**0.002**
**Eosinophils ($x10^3$/µL)**	0.22 (0 - 1.58)	N/A	0.18 (0 - 4.91)	0.423
**Time (days) to peak ALT (U/L), median (range)**	0 (0 - 77)	1 (0 - 54)	2 (0 - 44)	0.055
**Grade 4 injury (peak ALT ≥ 800 U/L), n (%)**	0	55 (31%)	9 (11%)	**<0.0001**
**Total bilirubin >3.6 mg/dL, n (%)**	5 (13%)	6 (3.4%)	9 (11%)	**0.017**
**DILIN severity score 3+, n (%)**	5 (13%)	11 (6%)	12 (15)	0.058

	**Cholestatic (n=39)**	**Hepatocellular (n=179)**	**Mixed (n=80)**	**P value**
**IEGW score moderate or severe pooled*, n (%)**	7 (18%)	14 (7.8%)	14 (18%)	**0.036**
**IEGW score severe, n (%)**	0	0	3 (3.8%)	**0.035**

* As only 3 patients in the entire cohort met IEGW severe criteria based on peak lab values, a pooled comparison of patients manifesting either moderate or severe injury versus mild injury was performed.

Abbreviations: ALT – alanine aminotransferase; ALP – alkaline phosphatase; AST – aspartate aminotransferase; DILIN – Drug Induced Liver Injury Network; GGT – gamma-glutamyl transferase; IEGW – International DILI Expert Working Group.

Supplemental Table 2: Outcomes stratified by R-value based on peak ALT and peak ALP.

	**Cholestatic (n = 39)**	**Hepatocellular (n = 179)**	**Mixed (n = 80)**	**P-value**
**Normalization of ALT (≤ 40 U/L), n (%)**	36 (92%)	170 (95%)	76 (95%)	0.704
**Time (days) to normalization of ALT, median (range)**	28 (6 - 175)	42 (7 - 301)	32 (6 - 180)	0.165
**Normalization of ALP (≤ 120 U/L), n (%)**	29 (74%)	N/A	70 (88%)	0.072
**Time (days) to normalization of ALP, median (range)**	42 (15 - 614)	N/A	30 (4 - 462)	**0.003**
**Improvement in ALT (≤ 100 U/L), n (%)**	38 (97%)	178 (99%)	79 (99%)	0.221
**Time (days) to improvement in ALT, median (range)**	15 (2 - 142)	17 (3 - 171)	16 (3 - 118)	0.635
**Improvement in ALP (≤ 300 U/L), n (%)**	36 (92%)	N/A	77 (96%)	0.392
**Time (days) to improvement in ALP, median (range)**	18 (7 - 63)	N/A	5 (1 - 381)	0.192

Abbreviations: ALT – alanine aminotransferase; ALP – alkaline phosphatase.

Supplemental Table 3 – Slow responders defined by time to ALT normalization (≤ 40 U/L) or ALT improvement (≤ 100 U/L) exceeding 100 days, stratified by liver injury pattern.

		**Time to ALT ≤ 40 U/L, normal responders (n = 259)**	**Time to ALT ≤ 40 U/L, slow responders (n = 39)**	**P value**	**Time to ALT ≤ 100 U/L, normal responders (n = 289)**	**Time to ALT ≤ 100 U/L, slow responders (n = 9)**	**P value**
**Total bilirubin at time of diagnosis (median, range)**		0.7 (0.1 - 21.3)	0.9 (0.2 - 6.1)	0.16	0.7 (0.1 - 21.3)	0.9 (0.3 - 2.8)	0.748
**Liver injury pattern, n (%)**	**Cholestatic (n = 39)**	34 (87%)	5 (13%)	0.38	38 (97%)	1 (3%)	1.00
	**Hepato-cellular (n = 179)**	152 (85%)	27 (15%)		173 (97%)	6 (3%)	
	**Mixed (n = 80)**	73 (91%)	7 (9%)		78 (98%)	2 (3%)	
**Hospitalized, n (%)**		148 (57%)	22 (56%)	0.93	165 (57%)	5 (56%)	1.00
**Steroid refractory, n (%)**		65 (25%)	19 (49%)	**0.002**	79 (27%)	5 (56%)	0.123

Abbreviations: ALT – alanine aminotransferase.

Supplemental Table 4 – Slow responders defined by time from peak ALT or ALP to normalization exceeding 100 days, stratified by liver injury pattern. Outcomes for the hepatocellular group are based on ALT normalization (≤ 40 U/L) while outcomes for the cholestatic and mixed groups are based on ALP normalization (≤ 120 U/L).

	**Cholestatic**<br>**(n = 39)**	**Hepatocellular**<br>**(n= 179)**	**Mixed**<br>**(n = 80)**	**P-value**
**Time to normalization of ALT (≤ 40 U/L) greater than 100 days, n (%)**	4 (10%)	21 (12%)	3 (3.8%)	0.124
**Time to normalization of ALP (≤ 120 U/L) greater than 100 days, n (%)**	14 (34%)	N/A	6 (7.5%)	**0.0001**
**Steroid refractory, n (%)**	0	11 (52%)	2 (33%)	**0.002**
**Liver biopsy¸ n (%)**	2 (14%)	12 (57%)	2 (33%)	**0.03**
**Hospitalized, n (%)**	4 (29%)	11 (52%)	5 (83%)	0.081
**Death, n (%)**	3 (21%)	7 (33%)	4 (67%)	0.210

Abbreviations: ALT – alanine aminotransferase; ALP – alkaline phosphatase.

Supplemental Table 5 – Outcomes among patients with 'Chronic DILI' defined by failure to achieve ALT ≤ 40 (for hepatocellular group) or ALP ≤ 120 (for cholestatic, mixed groups) by 6 months after diagnosis of immunotherapy-induced hepatoxicity. Outcomes for the hepatocellular group are based on ALT normalization (≤ 40 U/L) while outcomes for the cholestatic and mixed groups are based on ALP normalization (≤ 120 U/L).

	**Cholestatic (n = 39)**	**Hepatocellular (n = 179)**	**Mixed (n = 80)**	**P-value**
**Time to normalization of ALT (≤ 40 U/L) greater than 6 months, n (%)**	1 (2.6%)	5 (2.8%)	1 (1.3%)	0.749
**Time to normalization of ALP (≤ 120 U/L) greater than 6 months, n (%)**	10 (26%)	N/A	2 (2.5%)	**0.0002**
**Steroid-refractory, n (%)**	0	4 (80%)	2 (100%)	**0.0006**
**Liver biopsy, n (%)**	1 (10%)	3 (60%)	1 (50%)	0.079
**Hospitalized, n (%)**	3 (30%)	3 (60 %)	2 (100%)	0.205
**Death, n (%)**	1 (10%)	2 (40%)	1 (50%)	0.181

Abbreviations: ALT – alanine aminotransferase; ALP – alkaline phosphatase.

Supplemental Table 6 – Outcomes by DILIN severity score (3+ versus pooled 1+ or 2+)* and by International DILI Expert Working Group (IEGW) severity score (moderate versus mild) at the time of hepatotoxicity diagnosis.

	**DILIN 3+ (n = 18)**	**DILIN 1+/2+ (n = 279)**	**P value**	**IEGW moderate (n = 20)**	**IEGW mild (n = 277)**	**P value**
**ALT improved to ≤ 40 U/L, n (%)**	17 (94%)	264 (95%)	1.000	19 (95%)	262 (95%)	1.000
**Time (days) to ALT ≤ 40 U/L, median (range)**	49 (14 - 192)	39 (7 - 308)	0.254	50 (14 - 192)	38 (7 - 308)	0.188
**ALT improved to ≤ 100 U/L, n (%)**	18 (100%)	276 (99%)	1.000	20 (100%)	274 (99%)	1.000
**Time (days) to ALT ≤ 100 U/L, median (range)**	31 (5 - 126)	20 (3 - 176)	0.148	31 (5 - 126)	20 (3 - 176)	0.116
**Steroid refractory, n (%)**	8 (44%)	76 (27%)	0.174	8 (40%)	76 (27%)	0.228
**Liver biopsy**	7 (39%)	95 (34%)	0.675	7 (35%)	95 (34%)	0.949
**Death during follow-up, n (%)**	8 (44%)	119 (43%)	0.882	9 (45%)	118 (43%)	0.834
**Time (days) to death, median (range)**	232.5 (74 - 981)	311 (7 - 1774)	0.739	238 (74 - 981)	309 (7 - 1774)	0.966
**Hospitalized, n (%)**	N/A	N/A		18 (90%)	152 (55%)	**0.002**

*As hospitalization is a component of DILIN severity scoring, rates of hospitalization were not compared.

Abbreviations: ALT – alanine aminotransferase; ALP – alkaline phosphatase; DILIN – Drug Induced Liver Injury Network; IEGW – International DILI Expert Working Group.

Supplemental Table 7 – Outcomes stratified by the presence or absence of bile duct damage on histologic analysis.

	**Bile duct damage present (n = 29)**	**Bile duct damage absent (n = 73)**	**P value**
**ALT improved to ≤ 40 U/L, n (%)**	29 (100%)	71 (97%)	1.000
**Time (days) to ALT ≤ 40 U/L, median (range)**	44 (14 - 196)	49 (10 - 298)	0.698
**ALT improved to ≤ 100 U/L, n (%)**	29 (100%)	72 (99%)	1.000
**Time (days) to ALT ≤ 100 U/L, median (range)**	19 (7 - 147)	27 (3 - 176)	0.446
**Steroid refractory, n (%)**	12 (41%)	34 (47%)	0.634
**Hospitalized, n (%)**	17 (59%)	54 (74%)	0.128
**Death during follow-up, n (%)**	12 (41%)	32 (44%)	0.821
**Time (days) to death, median (range)**	450.5 (54 - 1040)	363 (46 - 1774)	0.549

Abbreviations: ALT – alanine aminotransferase.

Supplemental Table 8: Histopathology findings of patients with cholestatic injury who underwent liver biopsy.

Study ID	Portal inflammation	Bile duct inflammation	Ductular reaction	Bile duct loss	Cholestasis	Granulomas (non-necrotizing)	Centrilobular necrosis	Lobular inflammation	Periportal inflammation	Interface activity	Histiocyte aggregates	Endothelitis	Inflammatory cells	Fibrosis
**35**	2	2	0	0	0	1	0	1	0	0	1	0	L2, E1, N1, H1, P1	Portal
**53**	1	1	0	0	0	0	0	0	1	0	0	0	L2, H1, E1	None
**59**	2	0	0	0	0	2	0	1	0	1	0	3	L2, N1, E1	Portal
**61**	3	3	0	0	0	0	3	3	3	0	0	0	L3, P1, E1, N1, H1	Pericellular-mild
**108**	2	1	1	0	0	0	1	2	2	0	1	1	H3, L3, N1	None
**114**	1	1	0	0	0	2	0	0	0	0	0	0	L1	None
**116**	0	0	0	0	0	0	1	0	0	0	0	0	N/A	Focal portal
**150**	1	1	1	0	0	0	1	1	0	0	0	0	L1, H1	Patchy sinusoidal
**164**	0	0	0	0	0	0	0	0	1 - 2	0	0	0	L2, H1, E1	None
**183**	2	3	0	0	0	0	0	2	0	0	3	0	H3, L3, E1, N1	Periportal
**193**	1	1	1	0	0	0	0	1	0	0	0	0	L1	None
**196**	2	2	0	0	0	0	0	1	0	0	0	0	M2, E1, N1	Portal
**298**	2	2	1	0	1	3	0	0	1	0	1	0	L2, H2	Bridging

Table Legend: 0 = none, 1 = mild, 2 = moderate, 3 = severe. E = eosinophils, L = lymphocytes, M = monocytes, N = neutrophils, H = histiocytes, P = plasma cells.

Supplemental Table 9: Summary of MRCP findings performed during follow up for patients with either cholestatic or mixed liver injury.

	**Cholestatic (n = 39)**	**Mixed (n = 80)**	**P-value**
**MRI/MRCP obtained during follow up, n (%)**	14 (36%)	17 (21%)	0.088
**Time (days) to MRCP, median (range)**	3.5 (0 – 181)	7 (0 – 850)	0.289
**No biliary abnormalities seen, n (%)**	12 (86%)	12 (71%)	0.412
**Extra-hepatic biliary ductal involvement only, n (%)**	2 (14%)	3 (18%)	1.000
**Intra-hepatic biliary ductal involvement only, n (%)**	1 (7.1%)	5 (29%)	0.185
**Both intra- and extra-hepatic biliary ductal involvement, n (%)**	1 (7.1%)	3 (18%)	0.608
**Biliary ductal dilatation, n (%)**	2 (14%)	5 (29%)	0.412
**Biliary duct stricture, n (%)**	0	0	N/A
**Biliary duct thickening, n (%)**	0	1 (5.9%)	1.000

Abbreviations: Magnetic resonance cholangiopancreatography (MRCP).

Supplemental Figure 1 – Survival analysis by liver injury pattern as determined by R-value for: A) time to ALT ≤ 40 U/L (hepatocellular) or time to ALP ≤ 120 U/L (mixed/cholestatic groups), adjusted for ALT (hepatocellular) or ALP (mixed/cholestatic) at the time of ICI hepatotoxicity diagnosis ($p < 0.0001$); B) time to ALT ≤ 100 U/L (hepatocellular) or time to ALP ≤ 300 U/L (mixed/cholestatic groups), adjusted for ALT (hepatocellular) or ALP (mixed/cholestatic) at the time of ICI hepatotoxicity diagnosis ($p = 0.0003$).

Solid line: Cholestatic injury pattern; Dashed line: Mixed injury pattern; Dotted line: Hepatocellular injury pattern. Patients who died prior to improvement to ALT ≤ 40 U/L or ≤ 100 U/L, or ALP ≤ 120 or ≤ 300 U/L were censored from analysis.

Abbreviations: ALT – alanine aminotransferase; ALP – alkaline phosphatase.

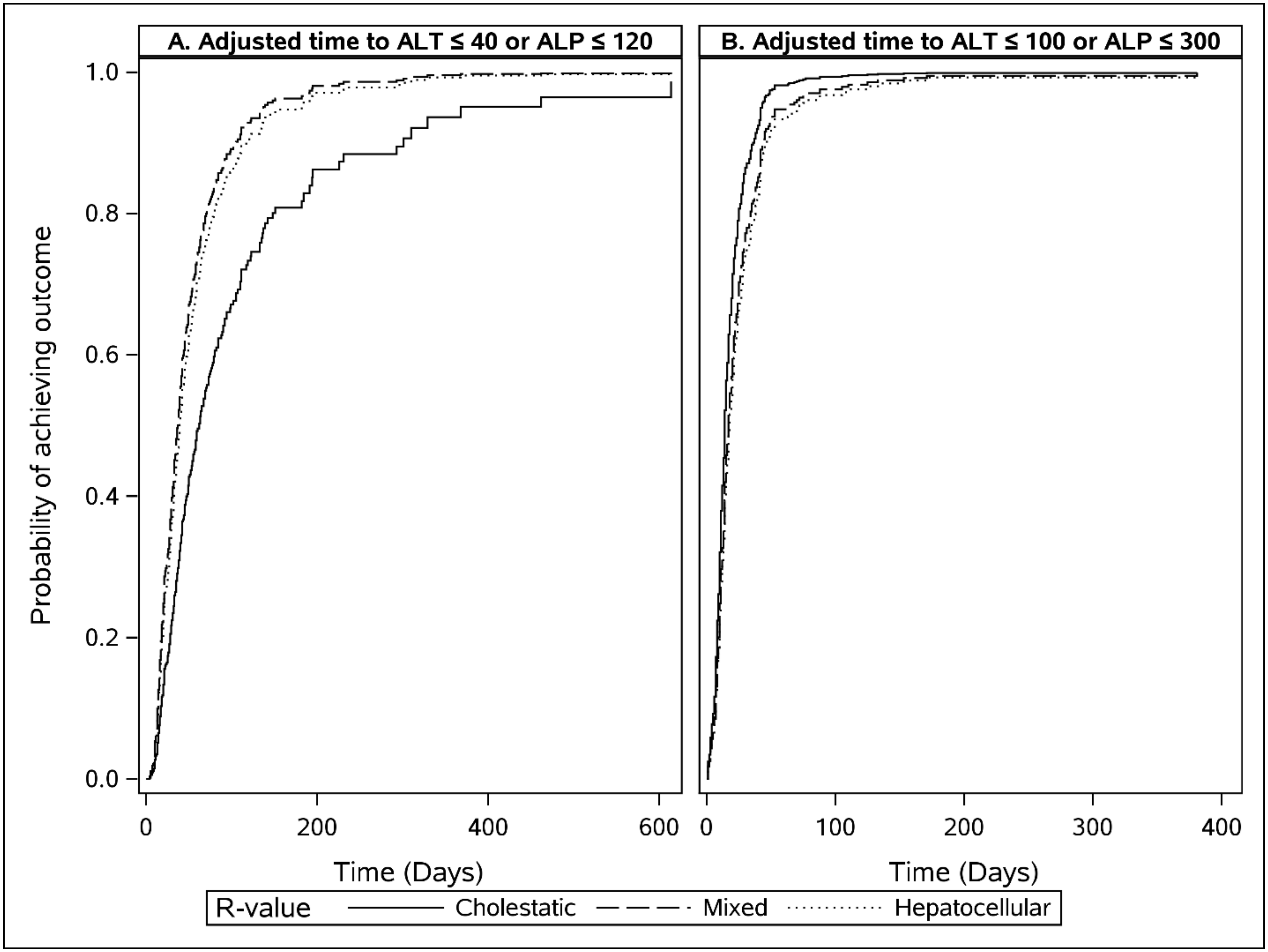
A. Adjusted time to ALT ≤ 40 or ALP ≤ 120
B. Adjusted time to ALT ≤ 100 or ALP ≤ 300
Probability of achieving outcome
1.0
0.8
0.6
0.4
0.2
0.0
0
200
400
600
0
100
200
300
400
Time (Days)
Time (Days)
R-value
Cholestatic
Mixed
Hepatocellular
