# Supplementary material for: Liver test pattern and histologic bile duct injury do not predict response to treatment of immunotherapy hepatotoxicity
Source: Hepatol Commun. 2026 Mar 26;10(4):e0922. doi: 10.1097/HC9.0000000000000922 (PMC13671605; doi:10.1097/HC9.0000000000000922)
Supplement: Supplementary file 2 [file hc9-10-e0922-s002.docx]

STROBE Statement—Checklist of items that should be included in reports of ***cohort studies***

|  | Item No | Recommendation |
| --- | --- | --- |
| **Title and abstract** | 1 | (*a*) Indicate the study’s design with a commonly used term in the title or the abstract  “Retrospective cohort study” named in Abstract Methods section. |
|  |  | (*b*) Provide in the abstract an informative and balanced summary of what was done and what was found  See page 2 of manuscript for abstract |
| Introduction | | |
| Background/rationale | 2 | Explain the scientific background and rationale for the investigation being reported  Paragraph 1 of Introduction (page 3, manuscript) |
| Objectives | 3 | State specific objectives, including any prespecified hypotheses  Paragraph 2 of Introduction (page 3, manuscript) |
| Methods | | |
| Study design | 4 | Present key elements of study design early in the paper  Section 2.1, “Patient data and study design” in Methods (page 3, manuscript) |
| Setting | 5 | Describe the setting, locations, and relevant dates, including periods of recruitment, exposure, follow-up, and data collection  Section 2.1, “Patient data and study design” in Methods (page 3, manuscript) |
| Participants | 6 | (*a*) Give the eligibility criteria, and the sources and methods of selection of participants. Describe methods of follow-up  Section 2.1, “Patient data and study design” in Methods (page 3, manuscript) |
|  |  | (*b*) For matched studies, give matching criteria and number of exposed and unexposed – N/A |
| Variables | 7 | Clearly define all outcomes, exposures, predictors, potential confounders, and effect modifiers. Give diagnostic criteria, if applicable Sections 2.2-2.3, “Patient stratification,” “Primary and secondary outcomes” in Methods (page 4-5, manuscript) |
| Data sources/ measurement | 8* | For each variable of interest, give sources of data and details of methods of assessment (measurement). Describe comparability of assessment methods if there is more than one group – Sections 2.2-4, “Patient stratification,” “Primary and secondary outcomes,” “Demographic data” in Methods (page 4-5, manuscript) |
| Bias | 9 | Describe any efforts to address potential sources of bias – Sections 2.1-3, “Patient data and study design,” “Patient stratification,” “Primary and secondary outcomes” in Methods (page 3-5, manuscript) |
| Study size | 10 | Explain how the study size was arrived at – section 2.1 patient data and study design, Methods (page 3, manuscript) |
| Quantitative variables | 11 | Explain how quantitative variables were handled in the analyses. If applicable, describe which groupings were chosen and why – section 2.5 “Statistical analysis” in Methods (page 5-6, manuscript) |
| Statistical methods | 12 | (*a*) Describe all statistical methods, including those used to control for confounding |
|  |  | (*b*) Describe any methods used to examine subgroups and interactions |
|  |  | (*c*) Explain how missing data were addressed |
|  |  | (*d*) If applicable, explain how loss to follow-up was addressed |
|  |  | (*e*) Describe any sensitivity analyses  Statistical methods, including handling of missing data, subgroup analyses, and multivariate analysis, are described in section 2.5 “Statistical analysis” in the methods section of the manuscript (page 5-6) |
| Results | | |
| Participants | 13* | (a) Report numbers of individuals at each stage of study—eg numbers potentially eligible, examined for eligibility, confirmed eligible, included in the study, completing follow-up, and analysed |
|  |  | (b) Give reasons for non-participation at each stage |
|  |  | (c) Consider use of a flow diagram  Detailed inclusion/exclusion criteria is provided in a prior publication from this group, cited by the paper in paragraph 1 of section 3.1, “Baseline data,” in the Results (page 6) |
| Descriptive data | 14* | (a) Give characteristics of study participants (eg demographic, clinical, social) and information on exposures and potential confounders  Descriptive data summarized in Results section 3.1, “Baseline Data” and Table 1 |
|  |  | (b) Indicate number of participants with missing data for each variable of interest  Missing data addressed in Methods section 2.5, “Statistical analysis” |
|  |  | (c) Summarise follow-up time (eg, average and total amount)  Follow up time summary provided in Methods section 2.4, “Demographic data” |
| Outcome data | 15* | Report numbers of outcome events or summary measures over time  Outcomes summarized in Results sections 3.3-3.7, and in Table 3 |
| Main results | 16 | (*a*) Give unadjusted estimates and, if applicable, confounder-adjusted estimates and their precision (eg, 95% confidence interval). Make clear which confounders were adjusted for and why they were included  Unadjusted and adjusted estimates are provided for the primary outcome in Figure 1 |
|  |  | (*b*) Report category boundaries when continuous variables were categorized  Median and range are provided for continuous non-normal variables; mean and standard deviation provided for continuous normal data, summarized in Tables 1-3. |
|  |  | (*c*) If relevant, consider translating estimates of relative risk into absolute risk for a meaningful time period – Not applicable for this study. |
| Other analyses | 17 | Report other analyses done—eg analyses of subgroups and interactions, and sensitivity analyses  Subgroup analyses summarized in Results sections 3.3-3.7, with summarized data in Table 3, and Figures 2 and 3. Additional subgroup analysis summaries provided in the Supplementary materials. |
| Discussion | | |
| Key results | 18 | Summarise key results with reference to study objectives – Paragraphs 1-3 of Discussion section. |
| Limitations | 19 | Discuss limitations of the study, taking into account sources of potential bias or imprecision. Discuss both direction and magnitude of any potential bias – Paragraph 7 of Discussion section details study limitations |
| Interpretation | 20 | Give a cautious overall interpretation of results considering objectives, limitations, multiplicity of analyses, results from similar studies, and other relevant evidence – Paragraphs 2-8 of Discussion section compares our results with existing evidence. |
| Generalisability | 21 | Discuss the generalisability (external validity) of the study results – Discussed in the strengths and limitations sections of the Discussion (page 12-13, manuscript) |
| Other information | | |
| Funding | 22 | Give the source of funding and the role of the funders for the present study and, if applicable, for the original study on which the present article is based – No funding source for this study, as is stated on page 1 of the manuscript. |

*Give information separately for exposed and unexposed groups.

**Note:** An Explanation and Elaboration article discusses each checklist item and gives methodological background and published examples of transparent reporting. The STROBE checklist is best used in conjunction with this article (freely available on the Web sites of PLoS Medicine at http://www.plosmedicine.org/, Annals of Internal Medicine at http://www.annals.org/, and Epidemiology at http://www.epidem.com/). Information on the STROBE Initiative is available at http://www.strobe-statement.org.
